# Second-Generation Androgen Receptor Antagonists as Hormonal Therapeutics for Three Forms of Prostate Cancer

**DOI:** 10.3390/molecules25102448

**Published:** 2020-05-24

**Authors:** Pravien Rajaram, Alyssa Rivera, Kevin Muthima, Nicholas Olveda, Hubert Muchalski, Qiao-Hong Chen

**Affiliations:** Department of Chemistry, California State University, Fresno, CA 93740, USA; pravien@mail.fresnostate.edu (P.R.); alyssamrivera@mail.fresnostate.edu (A.R.); muthima@mail.fresnostate.edu (K.M.); nicolveda08@mail.fresnostate.edu (N.O.)

**Keywords:** androgen receptor, prostate cancer, enzalutamide, apalutamide, darolutamide

## Abstract

Enzalutamide is the first second-generation nonsteroidal androgen receptor (AR) antagonist with a strong binding affinity to AR. Most significantly, enzalutamide can prolong not only overall survival time and metastatic free survival time for patients with lethal castration-resistant prostate cancer (CRPC), but also castration-resistant free survival time for patients with castration-sensitive prostate cancer (CSPC). Enzalutamide has thus been approved by the US Food and Drug Administration (FDA) for the treatment of both metastatic (in 2012) and non-metastatic (in 2018) CRPC, as well as CSPC (2019). This is an inspiring drug discovery story created by an amazing interdisciplinary collaboration. Equally important, the successful clinical use of enzalutamide proves the notion that the second-generation AR antagonists can serve as hormonal therapeutics for three forms of advanced prostate cancer. This has been further verified by the recent FDA approval of the other two second-generation AR antagonists, apalutamide and darolutamide, for the treatment of prostate cancer. This review focuses on the rational design and discovery of these three second-generation AR antagonists, and then highlights their syntheses, clinical studies, and use. Strategies to overcome the resistance to the second-generation AR antagonists are also reviewed.

## 1. Introduction

### 1.1. Prostate Cancer

Prostate cancer continues to be a main health concern due to the highest incidence and the second highest cancer-related death rate in American men. In 2020, estimates indicate about 21% of all new cancer cases will be attributed to prostate cancer, while over 33,000 deaths caused by prostate cancer are projected to occur in the United States [1]. The critical driving force for prostate cancer is the androgen receptor (AR)-regulated gene expression that is initiated by the binding of androgen to AR [2]. Consequently, the mainstay therapy for castration-sensitive prostate cancer (CSPC) since 1941 is androgen deprivation therapy (ADT). However, after the initial response to ADT for about 18 to 24 months, most CSPC will inevitably shift to castration resistant prostate cancer (CRPC) [3]. In the CRPC stage, prostate cancer continues to grow under extremely low levels of male hormone testosterone in serum. The majority of prostate cancer deaths in the United States are caused by late state (metastatic) CRPC (mCRPC). Within the past decade, several new treatments have been approved for three forms of prostate cancer: metastatic castration-sensitive prostate cancer (mCSPC), non-metastatic castration-resistant prostate cancer (nmCRPC), and metastatic castration-resistant prostate cancer (mCRPC). Table 1 lists the current treatments that have been approved by the US Food & Drug Administration (FDA) since 2004, according to the information published on the official website of the US FDA. Current treatments for prostate cancer can be classified into taxane-based chemotherapeutics, hormonal therapy, immunotherapy, and radiotherapy. As illustrated in Table 1, far more hormonal therapies than other categories have recently been approved by the US FDA for prostate cancer.

### 1.2. Hormonal Therapeutics

**Figure 1:**
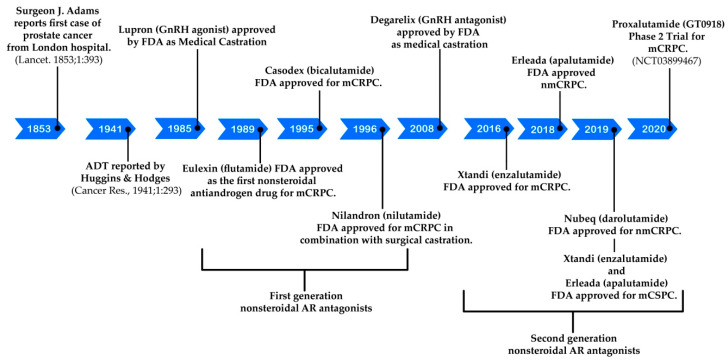
Timeline for the development of hormonal therapeutics for prostate cancer.

The timeline for the development of hormonal therapeutics for prostate cancer is illustrated in Figure 1. The pioneering hormonal therapeutic for prostate cancer is the well-known androgen deprivation therapy originally reported by Huggins and Hodges in 1941 [4]. At that point, orchiectomy (surgical castration) and administration of high dose of estrogen (non-surgical castration) were established to be two strategies to cut down the circulating testosterone to castrate (or near castrate) levels, resulting in appreciable biochemical response in a cohort of eight patients with metastatic prostate cancer. It was recognized by the Veterans Administration Cooperative Urological Research Group in 1970s that treatment of patients with advanced prostate cancer with high dose of estrogen led to good efficacy, but accompanying with enhanced mortality rate associated with cardiovascular complications [5]. The serious undesired effect of estrogen urged the scientists to search for a safer non-surgical castration strategy in 1980s. Encouraged by the finding that testicular production of testosterone can be indirectly controlled by long-lasting elevation of gonadotropin-releasing hormone (GnRH), GnRH agonists were designed and found to possess potential in suppressing prostate tumor growth in vivo and in clinical settings [6,7]. Synthetic GnRH agonists, e.g., goserelin (Zoladex) [8] and leuprolide (Lupron) [9], were developed as a replacement for estrogen as a better non-surgical castration strategy by the mid-1980s and have served as the centerpiece of ADT for CSPC since then. To conquer the testosterone surge as well as other side effects caused by GnRH agonists, the US FDA has approved degarelix (a GnRH antagonist) as an alternative medical castration for patients with advanced CSPC in 2008. As compared with GnRH agonists, degarelix can provide rapid suppression of prostate specific antigen (PSA) and testosterone so as to better control testosterone and prolong PSA progression-free survival [10]. Degarelix is, thus, a better non-surgical castration therapy for those CSPC patients at more advanced stages and with more apparent symptoms.

**Figure 2:**
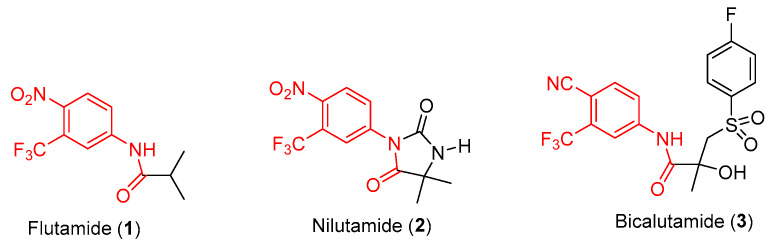
First generation of nonsteroidal AR antagonists.

On the other hand, examination of antiandrogen compounds as another alternative to estrogen castration was initiated in the late 1960s and early 1970s, leading to the development of three first generation nonsteroidal androgen antagonists, flutamide (**1**), nilutamide, (**2**) and bicalutamide (**3**), shown in Figure 2 [11]. These antiandrogen agents were revealed to competitively bind to the ligand-binding domain on androgen receptors. Monotherapy of bicalutamide (the one with the most extensive investigation) cannot offer better clinical benefit to patients with CSPC than ADT. The combination therapy of bicalutamide with ADT is widely used by CSPC patients owing to the greater safety profile than ADT alone, even though it does not grant significant overall survival benefit [12].

**Figure 3:**
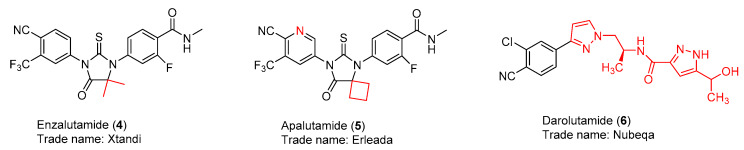
Second generation of nonsteroidal AR antagonists.

The clearer understanding of the structure and function of the androgen receptor revealed that the androgen receptor plays a pivotal role for not only CSPC but also CRPC [13]. This notation stimulated the successful design and discovery of three US FDA-approved second-generation androgen receptor antagonists, enzalutamide (**4**) [14], apalutamide (**5**) [15], and darolutamide (**6**) [16] (Figure 3). As illustrated in Table 2, enzalutamide (**4**) is now the first FDA-approved antiandrogen to treat three forms of advanced prostate cancer after the US FDA approval of enzalutamide (**4**) on 16 December 2019 for the treatment of metastatic castration-sensitive prostate cancer (mCSPC). The intriguing discovery stories of these three successful second-generation nonsteroidal AR antagonists are reviewed in this article. Their syntheses, clinical studies, and clinical use are highlighted as well. Current strategies to overcome the resistance to these AR antagonists are also summarized.

## 2. Discovery and Preclinical Studies

### 2.1. Enzalutamide *(**4**)* and Apalutamide *(**5**)*

**Figure 4:**
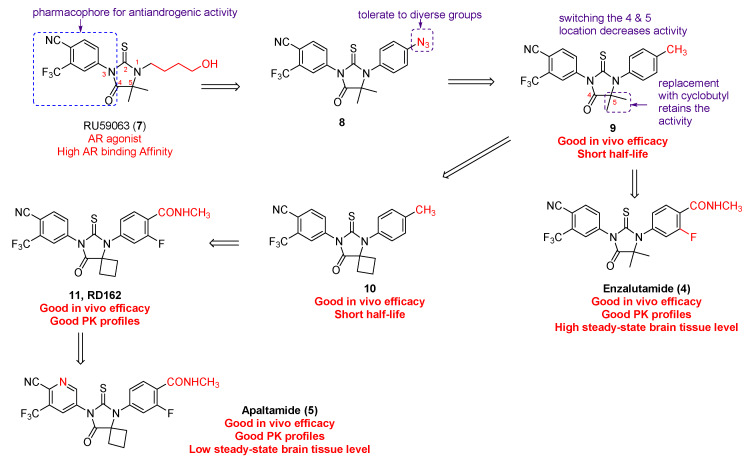
Discovery of enzalutamide and apalutamide.

Enzalutamide (**4**) and apalutamide (**5**) were discovered by the interdisciplinary collaboration of Sawyers/Jung groups, which was motivated by the notion that “growth of castration-resistant prostate cancer appears to depend upon continued androgen receptor signaling,” facilitated by the in vitro AR-overexpressing prostate cancer cell models, and benefited from a complementary collaboration [17]. As illustrated in Figure 4, RU59063 (**7**) was selected as the original lead compound because it is a potent and selective nonsteroidal AR agonist with high affinity for AR [18,19]. Enzalutamide (**4**) and apalutamide (**5**) were eventually identified as two lead candidates for preclinical development on the grounds of in vitro evaluation of their capability of agonistic and antagonistic activity of AR signaling in a castration-resistant LNCaP/AR prostate cancer cell model [20]. The in vitro relative luciferase activity and relative PSA level were measured using bicalutamide as positive control.

The structural modification started with substituting the ω-hydroxybutyl at *N*1 with azidoalkyl and azidoaryl groups in the light of the hypothesis that the small polar azido group might function as a bioisostere of the hydroxyl in RU59063 (**7**). The eastern side of RU59063 (**7**) was the first focus of the chemical manipulation probably due to the fact that the electron-deficient aromatic ring on the western side is a well-established pharmacophore for anti-androgenic activity [21]. Among the first set of analogues, compound **8** was identified as the optimal derivative that had higher binding affinity than bicalutamide. Further modification on compound **8** indicated that the 4-position of the *N*1-phenyl ring can accommodate several different groups without losing the desired activity. Compound **9** with a 4-methyl on the *N*1-phenyl ring was chosen for further structure-activity relationship studies, indicating that the location for the C-4 and C-5 on the thiohydantoin ring cannot be switched and that the geminal dimethyl group on the thiohydantoin ring can be substituted by the cyclobutyl ring in compound **10**. On the basis of abovementioned promising in vitro bioassay data, compounds **9** and **10** were moved forward for in vivo evaluation in a castrate mice model with LAPC4/AR or LNCaP/AR xenografts. Both compounds are more effective than bicalutamide in suppressing PSA secretion, but with a short half-life due to rapid clearance. Considering that electron-rich *N*1-phenyl ring and hydroxylation of the benzylic methyl, directly appending electron-withdrawing groups to the *N*1-phenyl ring led to the discovery of 3-fluoroamide analogues **11** (also called RD162) and enzalutamide (also called MDV3100). Both enzalutamide and RD162 (**11**) have greater (5–8 times) AR binding affinity relative to bicalutamide in the LNCaP/AR cell line with high level expression of wild-type AR. More importantly, their binding is specific to AR because only little or no binding to other nuclear receptors was observed [14].

Both enzalutamide and RD162 (**11**) possess not only excellent in vivo anti-tumor efficacy in the castrate mice model but also superb pharmacokinetic profile [14]. The in vivo pharmacokinetic properties of RD162 (**11**) were first evaluated in mice. The results showed that, after a 24-h oral treatment with a single 20 mg/kg dose, the plasma concentration (~23 µM) of RD162 (**11**) exceeds the concentration (~1–10 µM) necessary to block AR activity. The in vivo pharmacodynamic experiments suggest that RD162 (**11**) can significantly reduce AR transcriptional function and suppress LNCaP/AR tumor cell proliferation. The excellent in vivo efficacy of RD162 (**11**) in castration-resistant prostate tumor models was confirmed to be associated with AR suppression. This is because the effective dose for antitumor efficacy in the LNCaP/AR model is closely correlated with that for AR transcriptional activity as measured by luciferase imaging experiments. The fact that enzalutamide instead of RD162 (**11**) was chosen, at that moment, as the drug candidate for further preclinical studies is simply because enzalutamide can be prepared from an inexpensive starting material. Enzalutamide was successfully approved by FDA, but was found to be associated with seizure side effect caused by antagonizing GABA_A_ receptor in the central nervous system [22]. With the hope to find out a second-generation nonsteroidal AR antagonist with a high therapeutic index, apalutamide was later chosen for further preclinical investigation because of its lower steady-state brain tissue level in mice.

As shown in Figure 4, apalutamide (**5**, also named ARN-509) has very similar chemical structure to RD-162 (**11**) with only difference being the replacement of the *N*3-phenyl ring in RD162 (**11**) with a *N*3-pyridyl ring in apalutamide. Apalutamide possesses comparable in vitro activity to enzalutamide, but with greater anti-tumor efficacy in CRPC xenograft models and lower potential in causing seizure as an adverse effect in the central nervous system [14,23]. Specifically, enzalutamide (**4**) and apalutamide (**5**) were demonstrated to retain full antagonist activity in an AR overexpression setting and have a higher binding affinity of up to 10-fold for AR when compared to bicalutamide (**3**). Both of them compete with bicalutamide (**3**) for the same ligand-binding domain of AR. The selective binding of apalutamide (**5**) for AR over other nuclear hormone receptors was observed. Unlike the first-generation AR antagonists, both enzalutamide (**4**) and apalutamide (**5**) can interrupt multiple steps of the AR-signaling pathway, including the androgen binding to AR, nuclear translocation of AR, DNA binding, and coactivator recruitment. Good in vivo pharmacokinetic profiles, including good oral availability, long plasma half-life, and low systemic clearance, were found for both enzalutamide and apalutamide in mouse and dog models. However, apalutamide (**5**) has less chance than enzalutamide (**4**) to bind to plasma proteins, and 2-fold higher concentration of free apalutamide (**5**) was detected in mouse and human plasma. The in vivo pharmacodynamics studies of enzalutamide (**4**) and apalutamide (**5**) were carried out in a CRPC animal model with LNCaP/AR-luc xenograft tumors. Both of them have potent in vivo anti-tumor efficacy because they can significantly decrease androgen driven luciferase reporter-gene activity and reduce tumor volume compared to vehicle. However, apalutamide (**5**) only needs 10 to 30 mg/kg/d to reach maximum efficacy in the castrate mouse model with the LNCaP/AR xenografts, while enzalutamide (**4**) requires 30 to 100 mg/kg/d. Additionally, apalutamide exhibits antitumor activity in a CSPC xenograft model. The above-mentioned data provide preclinical proofs for further clinical development of apalutamide and enzalutamide for patients with CSPC and CRPC [14,23].

### 2.2. Darolutamide *(**6**)*

An AR transactivation screening of a group of nonsteroidal pyrazole-carboxamide and imidazole-carboxamide derivatives in an AR-HEK293 cell model in combination with a lead optimization process led to the discovery of darolutamide (**6**, also named ODM-201) [24]. Similar to enzalutamide (**4**) and apalutamide (**5**), darolutamide (**6**) is a full antagonist that has high affinity for AR in the AR overexpressing setting and suppresses nuclear translocation of AR. In contrast to enzalutamide (**4**) and apalutamide (**5**), darolutamide (**6**) possesses the following features [24]: (i) a different chemical scaffold that may bypass the side effects caused by enzalutamide (**4**) and apalutamide (**5**); (ii) antagonistic effects towards AR mutants AR(F876L), AR(W741L), and AR(T877A) that facilitate resistance to the first- and second-generation nonsteroidal AR antagonists; (iii) an inability to cross over the brain–blood barrier, suggesting a lower seizure risk than observed with enzalutamide; (iv) not increasing the concentration of serum testosterone in a mouse model; (v) having higher in vivo antitumor efficacy in the mouse model, and (vi) a shorter half-life (1.6 h vs 18.3 h for enzalutamide). Taken together, these promising preclinical results imply that darolutamide (**6**) is complementary to enzalutamide (**4**) and apalutamide (**5**) and that darolutamide (**6**) is an excellent addition to the family of the second generation of AR antagonists. However, higher dose and more frequent administration are recommended for darolutamide (**6**) due to its shorter half-life.

## 3. Syntheses

A detailed review of the development of synthetic approaches to enzalutamide, apalutamide, and darolutamide was recently published [25].

### 3.1. Synthetic Approaches to Enzalutamide *(**4**)* and Apalutamide *(**5**)*

**Scheme 1:**
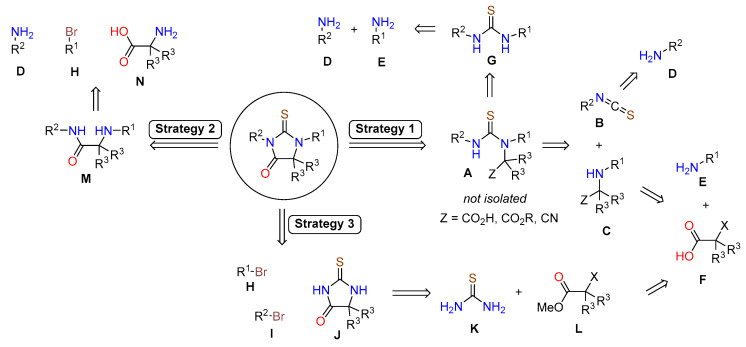
Overview of synthetic approaches to hydantoin-based enzalutamide and apalutamide.

Structures of enzalutamide (**4**) and apalutamide (**5**) are highly functionalized and offer multiple disconnection approaches to their synthesis. Each strategy is based on a key transformation of advanced intermediates and the bulk of the synthetic effort is spent on synthesizing those intermediates. The synthesis of the core structure of enzalutamide (**4**) and apalutamide (**5**) has been accomplished using three main strategies, which are presented in Scheme 1. Strategy 1 and Strategy 2 construct the thiohydantoin core toward the end of the synthesis, whereas Strategy 3 begins with the formation of thiohydantoin and the aromatic rings are added later. Regardless of the strategy, the assembly of the final drug begins from similar advanced aryl intermediates. Thus, the majority of process development was focused on preparation of aniline derivatives **D** and **E**, and aryl halides **H** and **I**.

#### 3.1.1. Strategy 1: Cyclization of Isothiocyanate

The first strategy is based on tandem condensation–cyclization cascade initiated by addition of isothiocyanate **B** to the α-amino acid derivative **C**, which delivers the thiourea intermediate **A** (Scheme 1). The compound **A** is not isolated, the nitrogen and the pendant carboxylic acid derivative (Z = CO_2_H, CO_2_R, or CN) react to form the thiohydantoin core. Thus, the key intermediates in this approach are isothiocyanate **B** and carboxylic acid intermediate **C**.

**Scheme 2:**
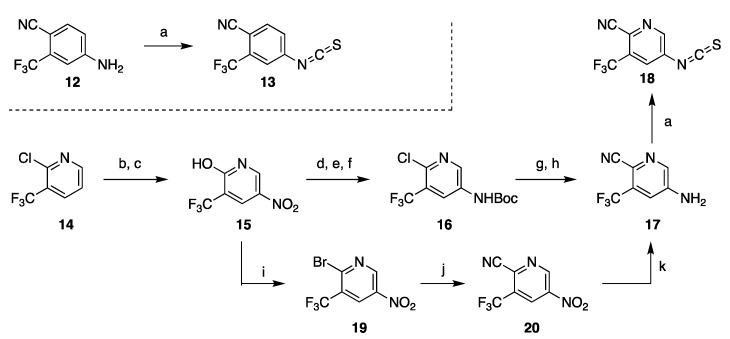
Synthesis of isothiocyanate fragments of enzalutamide and apalutamide. Reaction conditions: (a) CSCl_2_, H_2_O; (b) AcOH, H_2_O, reflux; (c) HNO_3_, H_2_SO_4_, 90 °C; (d) POCl_3_, PCl_5_, 110–120 °C; H_2_, (e) Raney Ni, THF; (f) Boc_2_O, pyridine, DMAP, rt; (g) KCN, CuCN, phenantroline, DMA, 110 °C; (h) TFA, CH_2_Cl_2_; (i) POBr_3_, PBr_3_, Br_2_, 90–110 °C; (j) CuCN, phenantroline, CH_3_CONMe_2_, 160 °C; (k) AcOH, Fe.

Sawyers and Jung disclosed the synthesis of **13** from aniline **12** and thiophosgene (Scheme 2) [17,26,27,28], to which alternative approaches that avoid use of thiophosgene were later developed [29,30,31,32]. The intermediate **12** needed for enzalutamide is commercially available. However, the aniline precursor of isothiocyanate fragment **18** needed for preparation of apalutamide had to be synthesized and proved to be a major challenge typical of heterocyclic amines containing both electron-rich and electron poor substituents. All published approaches to **17** vary in the order and method in which the substituents are added to 2-chloro-3-(trifluoromethyl)pyridine (**14**). The initially disclosed synthesis uses nitration–reduction sequence to install the amine functional group (Scheme 2). This strategy required uneconomical functional group interconversion (conversion **14** to hydroxypyridine **15**, then back to chloride **16** after nitration) as well as protection of the amine. The overall yield of this 7-step sequence is difficult to assess because yields for all steps were not reported. Alternately, cyanation can be accomplished prior to reduction of the nitro group and thus avoiding wasteful protection if a bromide is used instead of the chloride (steps i–k, Scheme 2) [33,34].

**Scheme 3:**
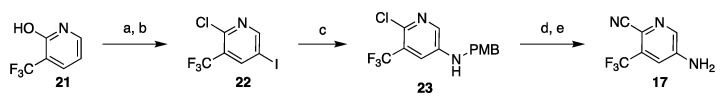
Nitration-free synthesis of the substituted pyridine intermediate **17**. Reaction conditions: (a) NIS, DMF, CH_3_CN, 80 °C; (b) POCl_3_, DMF, microwave 130 °C; (c) PMBNH_2_, Pd(OAc)_2_, BINAP, Et_3_N, CsCO_3_, toluene; (d) Zn(CN)_2_, Pd_2_(dba)_3_, dppf, DMF, 110 °C; (e) TFA, CH_2_Cl_2_.

Highly hazardous nitration at elevated temperature can be avoided by installing the amine via C–*N* cross-coupling reaction, although functional group interconversion (Cl to OH and back to Cl) remains as part of the route (Scheme 3). Initially developed conditions for the cross-coupling delivered **23** in low yield (40%) [33,34], but the reaction conditions were later improved to reliably generate the amine **23** in 71–85% yield [35,36]. Other routes to **17** were also disclosed, but they contain serious inefficiencies are less likely to be adopted on process scale [33,34].

**Scheme 4:**
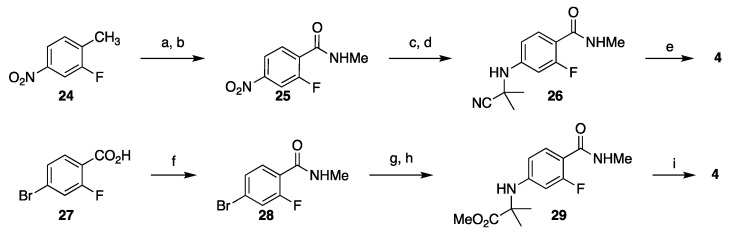
Synthesis of the amino nitrile **26** (top) and amino ester **29** (bottom) for the assembly of enzalutamide. Reaction conditions: (a) CrO_3_, H_5_IO_6_, CH_3_CN, CH_2_Cl_2_; (b) SOCl_2_, DMF, then MeNH_2_; (c) Fe, AcOH, EtOAc, reflux; (d) 2-cyano-2-hydroxypropane, MgSO_4_, EtOAc, 80 °C; (e) **13**, DFM, microwave 100 °C; (f) SOCl_2_, DMF (cat), 2-PrOAc, 60 °C, then MeNH_2_; (g) 2-aminoisobutyric acid, CuI, K_2_CO_3_, 2-acetylcyclohexanone, DMF, H_2_O, 105 °C; (h) MeI, K_2_CO_3_, DMF, H_2_O, 40 °C; **13**, DMSO, 2-PrOAc, 83–83 °C.

Synthesis of the advanced intermediate **C** (R = CN, Scheme 1) can be accomplished in several ways. In their initial route to enzalutamide, Sawyers and Jung reported preparation of **26** in 52% overall yield in a 4-step sequence which begins with oxidation of 2-fluoro-4-nitrotoluene (**24**) to the corresponding carboxylic acid [17], which was converted to an *N-*methyl amide **25** via the acid chloride intermediate. Reduction of the nitro group to an amine and addition to acetone cyanohydrin furnish α-amino nitrile **26**, which upon reaction with isothiocyanate **13** under microwave heating in DMF, gave enzalutamide (**4**). In another approach, amino ester **29** was prepared in 4 steps from 4-bromo-2-fluorobenzoic acid (**27**) in 64% yield by first converting the acid to the *N-*methyl amide followed by a S_N_Ar reaction with 2-aminoisobutyric acid and esterification. Although this route is shorter and the overall yield is slightly higher than the synthesis of nitrile **26**, on a process scale the likely starting material would be 4-bromo-2-fluorotoluene which would add one additional step and likely reduce the overall yield (Scheme 4).

**Scheme 5:**
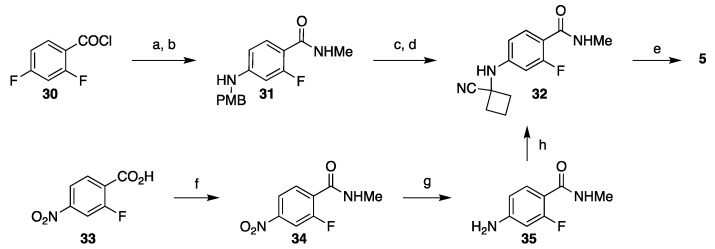
Two options for synthesis of amino nitrile **32** for the assembly of apalutamide. Reaction conditions: (a) MeNH_2_, THF; (b) PMBNH_2_, MeCN, microwave 190 °C; (c) TFA, CH_2_Cl_2_; (d) cyclobutanone, NaCN; (e) **18**, CSCl_2_, MeCONMe_2_, 80 °C, then HCl, MeOH; (f) Me_2_NH–HCl, CDI, Et_3_N, CH_2_Cl_2_, rt; (g) Fe, AcOH, EtOAc, reflux; (h) cyclobutanone, TMSCN, AcOH, 80 °C.

The amino nitrile fragment needed for assembly of apalutamide was prepared similarly in a 4-step sequence which begins with acylation of methyl amine with 2,4-difluorobenzoyl chloride (**30**) followed by S_N_Ar reaction with 4-methoxybenzyl amine under microwave conditions to give **31**. Acid-mediated deprotection and a Strecker reaction with cyclobutanone in the presence of sodium cyanide give amino nitrile **32** (Scheme 5, top). Although this route is relatively short, it will be difficult to implement at scale. The yield of the S_N_Ar reaction is low (40%) due to poor regioselectivity; the yield of the Strecker reaction was not reported. Additionally, use of cyanide at scale is challenging due to potential release of HCN. The company Hinova developed this route into a 3-step preparation (69%–82% yield) of **32** in which 2-fluoro-4-nitrobenzoic acid (**33**) was converted to a methyl amide followed by reduction of the nitro group and a Strecker reaction with cyclobutanone with TMSCN as cyanide source (Scheme 5, bottom) [37,38].

**Scheme 6:**
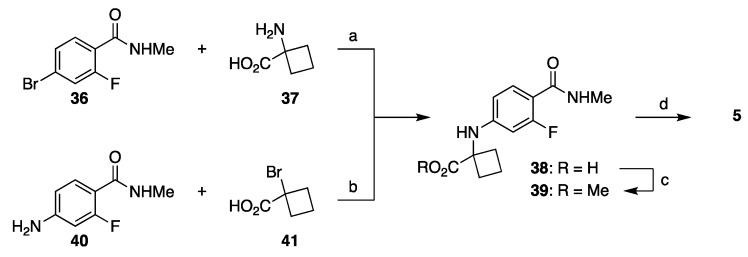
Cyclobutanecarboxylic acid-based approaches to the advanced intermediate **39** needed for assembly of apalutamide. Reaction conditions: (a) DMF, K_2_CO_3_, CuCl, H_2_O, 100–105 °C; (b) i-Pr_2_Net, Et_3_N, CH_2_Cl_2_, reflux; (c) MeI, DMF, K_2_CO_3_, rt; (d) **18**, DMSO, 2-PrOAc, 90 °C.

Several companies subsequently explored other carboxylic acid derivatives, with the most successful one being ester-based route shown in Scheme 6. Coupling between aryl bromide **36** and cyclobutane amino acid **37** gives amino acid derivative **38** (step a, Scheme 6) [39,40]; alternatively, acid **38** can be prepared from amine **40** and α-bromo acid **41** (step b, Scheme 6) [41]. Alkylation of the carboxylic acid **38** followed by a reaction with isothiocyanide **18** delivered apalutamide in 47% yield from **38**. It is important to note that condensation/cyclization cascade of esters and isothiocyanate delivers thiohydantoin in higher yield than the same reaction with the nitrile, but the higher yield comes with trade-offs. First, one additional step will be needed to convert the ester to amide (MeNH_2_, heat). Second, the cyclization reaction of isothiocyanate with esters produces alcohol by-product, which is reactive toward the isothiocyanate present in the reaction mixture. Therefore, excess of isothiocyanate has to be used in this step and at least one equivalent is ultimately lost to alcoholysis.

**Scheme 7:**
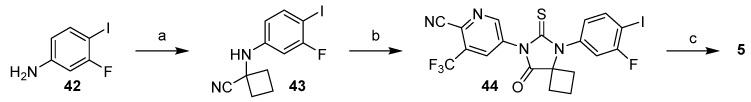
Synthesis of thiohydantoin core of apalutamide and late-stage amide formation. Reaction conditions: (a) cyclobutanone, NaCN, AcOH; (b) **17**, 1,1′-thiocarbonylbis(pyridine-2(1*H*)-one, toluene, 100 °C then HCl, EtOH, DMA, 70 °C; (c) *n-*C_5_H_11_MgBr, THF, CO_2_, then CDI, MeNH_2_, THF; or Pd(*t-*Bu_3_P)_2_, CO (5 bar), *i-*Pr_2_Net, MeNH_2_, THF, 60 °C; or Pd(OAc)_2_, dppf, *i-*Pr_2_NH, CO, MeOH, 60 °C, then MeNH_2_, MeOH.

Other iterations of Strategy 1 were also used in synthesis of apalutamide where the thiohydantoin core was constructed prior to installation of the methyl benzamide (Scheme 7) [42,43]. Halogenated aniline **42** was subjected to a Strecker reaction with cyclobutanone and the resulting amino nitrile **43** was reacted with isothiocyanate **13** formed in-situ from aniline **17** and 1,1′-thiocarbonylbis(pyridine-2(1*H*)-one (vide infra). Appending the methyl amide functional group can then be accomplished in several ways (Scheme 7, step c): (i) Grignard synthesis of carboxylic acid followed by CDI coupling with methyl amine; (ii) direct Pd-catalyzed amide formation with methylamine and carbon monoxide; or (iii) Pd-catalyzed carbonylative esterification followed by a conversion of the ester to the amide.

#### 3.1.2. Strategy 2: Late Stage Cyclization of Amino Amide

**Scheme 8:**
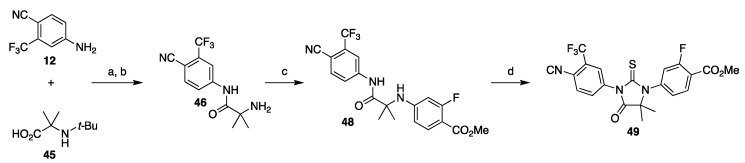
Synthesis of advanced intermediate ester **49** via late-stage formation of thiohydantoin. Reaction conditions: (a) CDI, DBU, *i-*Pr_2_NEt, THF, 55–65 °C; (b) HCl, 2-PrOH; (c) methyl 4-bromo-2-fluorobenzoate (**47**), acetylacetone, CuI, K_2_CO_3_, DMF, 120–130 °C; CSCl_2_, DMAP, THF, 40–50 °C.

The second strategy focuses on the late stage formation of thiohydantoin core through cyclization of the amino amide **M** with thiophosgene or its surrogate (Scheme 1, Strategy 2). Depending on the chosen disconnection, the amino amide **M** can be constructed by condensation of amine **D** with amino acid **N**, or aryl-aryl cross-coupling of the amine **D** with bromide **H**. An application of this strategy was reported by Meng and co-workers in their approach to enzalutamide, which started with a carbodiimide coupling of aniline **12** and protected amino acid **45**, affording amino amide **46** after deprotection (Scheme 8). Copper-catalyzed aryl amination with methyl 4-bromo-2-fluorobenzoate (**47**) affords amino amide **49**, which undergoes the cyclization reaction with thiophosgene in the presence of 8-fold excess of DMAP to provide ester **49** in 28% overall yield [44].

**Scheme 9:**
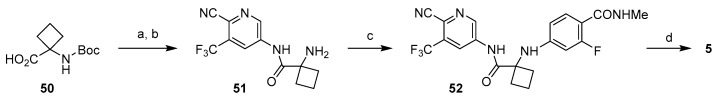
Synthesis of apalutamide via late-stage, thiophosgene-free cyclization. Reaction conditions: (a) **12**, CDI, DBU, *i-*Pr_2_NEt, THF, 60 °C; (b) HCl, 2-PrOH, 70 °C; (c) **28**, DMA, KOAc, CuBr, TMEDA, 120 °C; (d) 1,1′-thiocarbonylbis(pyridine2(1*H*)-one, DMAP, DMA, 90 °C.

As illustrated in Scheme 9, a modified version of this strategy was used in the synthesis of apalutamide. The key differences include more streamlined synthesis of the starting aniline **12**, use of Boc as protecting group for the amino acid **50**, and use of 1,1′-thiocarbonylbis(pyridine-2(1*H*)-one (or phenylthionochloroformate) as thiophosgene alternative for the formation of thiohydantoin core [45]. This route is a highly developed process that achieves synthesis of apalutamide in seven linear steps with only three purifications. Accounting for two-step synthesis of hydroxypyridine **15** (see Scheme 2), this route achieves preparation of apalutamide in nine linear steps.

#### 3.1.3. Strategy 3: Functionalization of Thiohydantoin

**Scheme 10:**
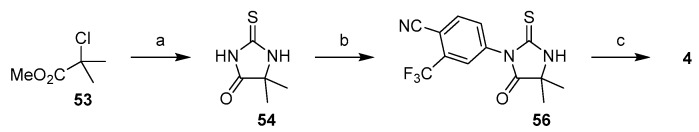
Synthesis of enzalutamide via functionalization of thiohydantoin. Reaction conditions: (a) thiourea, Et_3_N, DMF, 80–90 °C; (b) 4-bromo-3-(trifluoromethyl)-benzonitrile (**55**), NaH, DMF, rt; (c) **28**, NaH, DMF.

The third strategy focuses on the formation of thiohydanotin core first and then functionalization the nitrogens (Strategy 3, Scheme 1). In comparison to the two strategies outlined above, this one is relatively less developed. Nevertheless, it holds the most promise for large-scale synthesis of enzalutamide because it is still highly divergent and, even more importantly, avoids use of thiophosgene. The reported synthesis of enzalutamide using this approach begins with a reaction of methyl 2-chloroisobutyrate (**53**) with thiourea in the presence of triethylamine to give thiohydantoin **54**. Compound **54** then undergoes two S_N_Ar reactions. Deprotonation with NaH in DMF followed by addition of 4-bromo-3-(trifluoromethyl)benzonitrile (**55**) furnished compound **56**, which is deprotonated again and reacted with aryl bromide **28** to provide enzalutamide (Scheme 10) [32]. It should be noted that, at the time of this writing, methyl 2-chloroisobutyrate is not widely available from commercial sources and will have to be synthesized [46].

**Scheme 11:**
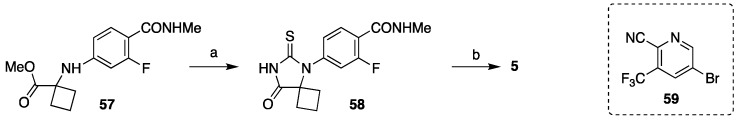
Synthesis of apalutamide using atom-economical formation of isothiohydantoin core. Reaction conditions: (a) KSCN, *i-*Pr_2_NEt, MeOH/H_2_O, reflux; (b) **59**, K_2_CO_3_, CuI, DMF, 100–110 °C.

A hybrid variant of this strategy and Strategy 1 were also used in synthesis of apalutamide [47]. The ester **57** was first reacted with potassium isothiocyanate to give thiohydantoin **58**, which was then coupled with aryl bromide **59** to give apalutamide. Both **57** and **58** can be purified by crystallization, which is a significant advantage for process development (Scheme 11).

### 3.2. Synthesis of Darolutamide

**Scheme 12:**
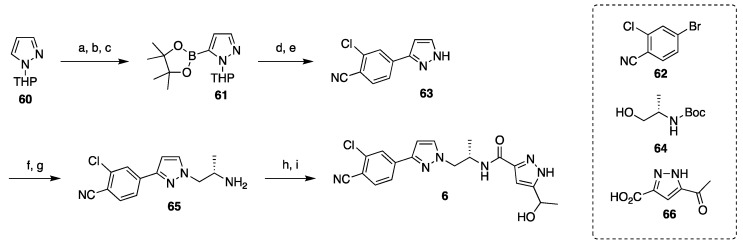
Synthesis of darolutamide. Reaction conditions: (a) n-BuLi, THF, toluene; (b) (i-PrO)_3_B; (c) pinacol, AcOH; (d) **62**, Pd(OAc)_2_, K_2_CO_3_, CH_3_CN/H_2_O, 70 °C; (e) aq HCl, MeOH, 10 °C; (f) **64**, PPh_3_, DIAD, EtOAc; (g) aq HCl, 45 °C; (h) **66**, T3P, EtOAc, i-Pr_2_NEt, 10 °C; (i) NaBH_4_, EtOH.

The development of synthesis of darolutamide was mostly focused on process improvements, such as isolation via crystallization, elimination of expensive and hazardous reagents, and the general strategy remained unchanged since the original disclosure (Scheme 12) [48,49,50,51]. The synthesis relies on cross-coupling and substitution chemistry with some protection/deprotection and functional group interconversions. First, a pinacol boronic ester is installed on the THP-protected pyrazole **60** by treatment with *n-*BuLi, then with triisopropylborane, and finally with pinacol. Palladium(II) acetate-catalyzed Suzuki cross-coupling of **61** with aryl bromide **62** delivers intermediate **63**. The nitrogen was deprotected with aqueous acid and reacted with *N-*protected (*S*)-(+)-2-amino-1-propanol (**64**) under Mitsunobu conditions followed by deprotection at low pH to give intermediate **65**. The synthesis of darolutamide diastereomers was completed by amide coupling of **65** with carboxylic acid **66** followed by reduction of the methyl ketone with NaBH_4_ in ethanol.

## 4. Clinical Studies and Use of Second Generation Nonsteroidal AR Antagonists

Only those clinical studies directly associated with the US FDA approval of enzalutamide, apalutamide, and darolutamide were highlighted in this review.

### 4.1. Enzalutamide

Enzalutamide (also named MDV3100; trade name: Xtandi) has been approved by the FDA in 2012, 2018, and 2019, respectively, for the treatment of mCRPC, nmCRPC, and mCSPC. Enzalutamide possesses a generally good safety profile and is now widely used as a standard-of-care for the treatment of three forms of prostate cancer. The clinical use of enzalutamide not only helps to better manage prostate cancer but also verifies that androgen receptor signaling continues to be one of critical driving forces for CRPC. Enzalutamide can be sequentially used with other therapeutic methods.

The earlier clinical trials of enzalutamide were initiated by its promising efficacy and drug-like properties collected from the preclinical CRPC models. The competitive AR binding capability and clinically effective antitumor activity, together with tolerable safety profile of enzalutamide, were verified in human by a phase I/II study that enrolled 140 patients with CRPC in both pre- and post-chemotherapy settings [52]. The maximal tolerated dose for enzalutamide was determined to be 240 mg/day and the recommended dose for the advanced clinical trials was identified to be 160 mg/day. In view of these inspiring early-stage clinical results, the first phase III trial of enzalutamide, named AFFIRM, has been started to scrutinize enzalutamide versus placebo in patients with mCRPC in a post-chemotherapy setting.

#### 4.1.1. Enzalutamide for mCRPC

With overall survival as its primary end point, AFFIRM aimed to assess whether enzalutamide can prolong the survival time of patients with mCRPC in a post-chemotherapy setting [53]. The clinical benefits derived from AFFIRM as demonstrated by the primary end points and the secondary end points were summarized in Table 3. The primary outcome from this phase III trials established enzalutamide as the first nonsteroidal AR antagonist with significant increase in patient’s overall survival time. The secondary outcome suggested that enzalutamide can appreciably slow cancer progression, reduce PSA response, and improve patient’s quality of life when compared to placebo. The major adverse effects of enzalutamide observed from AFFIRM are seizures, with 0.6% (five patients out of 800) of patients from the enzalutamide treatment group being reported to have a seizure. This study verified that AR and AR signaling continue to play a pivotal role for the progression of CRPC. Enzalutamide was thus quickly approved by the US FDA on 31 August 2012, for the treatment of late-stage mCRPC due to its capability of prolonging patient’s life.

Inspired by the positive results from AFFIRM and the greater benefit observed in chemotherapy-naïve patients in the phase I-II clinical trial [52], another phase III trial (named PREVAIL, ClinicalTrials.gov number, NCT01212991) of enzalutamide was designed to explore the possibility of extending the application of enzalutamide to patients with mCRPC before chemotherapy [54]. A total of 1717 patients with chemotherapy-naïve mCRPC were enrolled in this trial and randomized into enzalutamide treatment (872; 160 mg daily) group and placebo group (845). Radiographic progression-free survival and overall survival were used as the two primary end points. The clinical benefits brought by enzalutamide treatment in this study with respect to all primary and secondary end points are illustrated in Table 4. It can be concluded from these data that treatment with enzalutamide resulted in a noticeable decrease in risk of radiographic progression and death, as well as a marked delay in the need of chemotherapy. Due to the promising results from PREVAIL phase III trial, the clinical use of enzalutamide was therefore extended by the US FDA to chemotherapy-naïve patients with mCRPC on 10 September 2014.

#### 4.1.2. Enzalutamide for nmCRPC

The patients with nmCRPC are refractory to ADT treatment and at the onset of metastasis. It has therefore been recognized that new treatment strategies are urgently needed to reduce the risk for metastasis in men with nmCRPC in accompany with a short PSA doubling time. PROSPER phase III trial was designed to investigate whether enzalutamide can meet this need [55]. This study enrolled a total of 1401 patients with nmCRPC and a PSA doubling time no more than 10 months. A total of 933 patients received enzalutamide treatment (160 mg daily) in combination with ADT; while the remaining 468 patients were assigned to the placebo group with continued ADT. Metastasis-free survival was set as the primary end point. As illustrated in Table 5, enzalutamide treatment is superior to placebo with regards to the primary end point and most of the secondary end points. The clinical results from the PROSPER trial highlighted that the risk of progression to mCRPC or death in the enzalutamide treatment group has been lowered by 71% as compared with the placebo group. Because of the promising results from the PROSPER phase III trial, enzalutamide was approved by the US FDA, after Priority Review designation, on 13 July 2018, for the treatment of patients with nmCRPC. This approval extends the enzalutamide treatment to patients with nmCRPC, and makes enzalutamide the first FDA-approved oral medicine for both mCRPC and nmCRPC.

#### 4.1.3. Enzalutamide for mCSPC

Patients with mCSPC is defined as those who have metastatic prostate cancer that still responds to ADT. Up to 5% of annual prostate cancer incidences belong to mCSPC in the United States [56]. With ADT as the original standard of care, most of the patients with mCSPC inevitably progress to high-risk mCRPC in 1–3 years. To help meet the needs of this big group of patients, the ARCHES Phase III trial (ClinicalTrials.gov identifier: NCT02677896) [57] aimed to investigated whether enzalutamide in combination with ADT can prolong radiographic progression-free survival using ADT alone as control. The clinical outcomes from this trial pertaining to the primary end point (radiographic progression-free survival) and key secondary end points are summarized in Table 6. A conclusion can be drawn from these data that enzalutamide, plus ADT, demonstrate clinically significant improvement in efficacy by prolonging the radiographic progression-free survival while maintaining the safety level, as compared with ADT alone. The favorable results encouraged the US FDA to grant an extension of enzalutamide for the treatment of mCSPC on 16 December 2019.

### 4.2. Apalutamide

The promising preclinical data of apalutamide were well-mirrored by its phase I clinical trial [23,58]. Thirty men with mCRPC were enrolled in its first-in-human study. Apalutamide displayed AR inhibitory ability as evidenced by the 47% PSA response (defined as ≥50% reduction from baseline) at 12 weeks at all tested doses from 30 to 480 mg. The pharmacokinetic profile of oral-administrated apalutamide is linear and dose-dependent in the range of 30–480 mg. The rapid absorption was demonstrated by the fact that it can be measured in the plasma at 30 min after oral intake and that it can reach peak concentrations after 2–3 h. A half-life of 3–4 days was observed in the systematic circulation and most enrolled patients reached a steady-state concentration after 3 weeks of non-interrupted administration of apalutamide. A good safety profile observed from this clinical study further confirmed the high therapeutic index evaluated from the preclinical models. A daily dose of 240 mg was recommended for the follow-up clinical studies of apalutamide considering its dose to maximize the tumor regression in preclinical models, along with peak plasma concentration, safety profile, and efficacy in the phase I clinical study.

#### 4.2.1. Apalutamide for nmCRPC

The SPARTAN trial is a randomized, double blind and multicenter phase III study that evaluated the efficacy of apalutamide in nmCRPC. There were 1207 enrolled men with nmCRPC on ADT with a PSA doubling time of over 10 months randomized to 2:1 to receive apalutamide with ADT or placebo with ADT [59].

The first interim analysis, which concluded in May 2017, determined that there was statistical significance in metastasis free survival (MFS), progression free survival (PFS), time to metastasis and time to symptomatic progression (Table 7). Due to the compelling evidence shown, the safety monitoring committee recommended that the placebo group be allowed to receive the treatment in July 2017. The second interim analysis continued to 2019 to better characterize the effect of apalutamide. It was determined that the median MFS was 40.5 months for the treatment group and 16.2 months in the placebo group with a 95% confidence interval. The four-year survival rate of apalutamide was found to be 72% compared to 65% of the placebo group. When considering the patients that crossed over from placebo to treatment, the four-year survival rate remained at 72% for apalutamide [59].

Disease progression was the most frequent indicator for treatment discontinuation. Only 28% of the treatment group compared to 37% of control experienced cancer progression for discontinuation. Apalutamide treatment led to the extension of the second PFS rate by approximately 11.8 months versus placebo. The four-year second PFS rate for the treatment had a 19% difference when compared to the control [59]. Adverse effects were reported in 97% of patients in the treatment group and 94% of patents in the control group [59,60]. Median overall survival (OS) was not reached in either the treatment or placebo group, as the threshold of 427 deaths have not yet been reached as specified in the O’Brien-Fleming boundary [59].

Overall, apalutamide showed a quarter reduction for risk of death when compared to placebo as well as a higher subsequent life-prolongation, despite crossover. The resulting data for SPARTAN included statistical significance in improving MFS and time to symptomatic progression compared to placebo. The observations that apalutamide delays progression and death when combined with ongoing ADT suggests that the drug may be advantageous to high-risk nmCRPC patients. FDA immediately approved apalutamide for high-risk nmCRPC patients on 14 February 2018 [59].

#### 4.2.2. Apalutamide for mCSPC

Apalutamide was approved by the US FDA, after a priority review, on 17 September 2019, to extend its treatment from patients with nmCRPC to those with mCSPC based on the efficacy demonstrated by its TITAN (NCT02489318) phase III trial. This clinical trial was designed as a randomized, double blind and multicenter phase III study and aimed to assess the clinical efficacy of apalutamide in combination with ADT (surgical or medical castration) in patients with mCSPC. A study of 1052 men with mCSPC were randomized 1:1 to treatment and control groups. This involved patients regardless of disease volume and with a history of docetaxel treatment and treatment for localized prostate cancer. At the conclusion of the first interim analysis in November 2018, it was found that 68.2% of patients in the apalutamide and ADT group had a 24-month radiographic progression-free survival compared to 47.5% of the placebo group (Table 8). The overall survival, measured after 200 deaths, for 24-months is 82% for apalutamide group and 73% for the placebo group. The average time for second progression-free survival was also longer in the treatment group compared to that of the placebo group. Analysis of adverse effects between apalutamide and placebo did not differ significantly [61]. In summary, the TITAN trial revealed that apalutamide in combination with ADT resulted in life-prolongation and radiographic progression-free survival relative to placebo with ADT while also preserving quality of life for men with mCSPC [61].

#### 4.2.3. Indirect Comparison with Enzalutamide

In the lack of direct comparative studies, Chowdhury et al. conducted a matching-adjusted indirect comparison (MAIC) of the efficacy and quality of life of apalutamide to that of enzalutamide in nmCRPC using data collected from SPARTAN and PROSPER trials [62]. A total of 1171 patients were matched from the SPARTAN trial (n = 1207) to the PROSPER trial (n = 1401). Relative to enzalutamide, apalutamide was better tolerated based on adverse events with an overall decrease in fatigue, low appetite, hypertension, and nausea occurrences. It was also associated with a better health-related qualify of life based on the Functional Assessment of Cancer Therapy-Prostate score [63]. Additionally, the calculated hazard ratios for apalutamide versus enzalutamide were 0.77 for OS and 0.91 for MFS [62]. Based on these MAIC results, apalutamide demonstrates slightly better overall tolerability and is associated with slightly higher efficacy in nmCRPC patients.

### 4.3. Darolutamide

It has recently been recognized that early and effective suppression of AR signaling may serve as a good strategy to manage nmCRPC [64], which has been firmly supported by the obvious metastasis-free survival benefits reported in the PROPER trial for enzalutamide and the SPARTAN trial for apalutamide. The clinical phases I and II studies [65,66] suggest that darolutamide provides not only meaningful antitumor efficacy but also a favorable safety profile in clinical settings. ARAMIS phase III trial was thus conducted to further assess the treatment benefits and the possible adverse events of darolutamide in men with nmCRPC. A total of 1509 men with nmCRPC and a PSA doubling time no more than 10 months were enrolled in this trial and were randomized in a 2:1 ratio to receive darolutamide (600 mg twice daily) plus ADT or ADT alone. Metastasis free survival was employed as the primary end point, and the appearance of metastasis was judged by blinded and independent central imaging review. The therapeutic benefits of darolutamide were consistently and affirmatively judged by the primary end point and the entire secondary end points (Table 9). Darolutamide in combination with ADT was demonstrated to prolong metastasis-free survival by 22 months and to reduce the risk of metastasis or death by 59% when compared with ADT alone. The therapeutic benefits brought by darolutamide, enzalutamide, or apalutamide are generally similar in patients with nmCRPC. However, darolutamide exhibited a good safety profile in this phase III trial with approximately similar incidence of adverse events in the darolutamide treatment and placebo groups. The fact that darolutamide has less common adverse effects in the phase III trial relative to enzalutamide and apalutamide is associated with its low penetration of the blood-brain barrier as evidenced in preclinical studies. Darolutamide was approved by the US FDA on 30 July 2019, for the treatment of nmCRPC in line with ARAMIS phase III trial [16].

## 5. Mechanism of Action of the Second-Generation AR Antagonists

**Figure 5:**
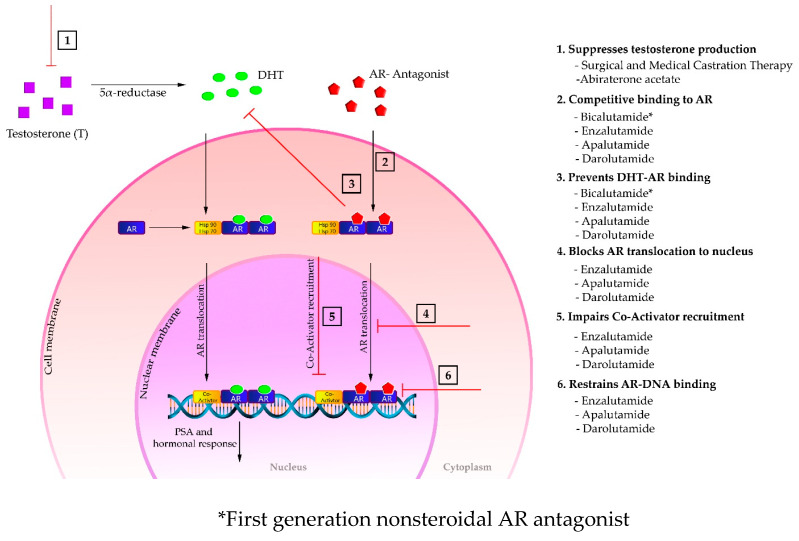
Mechanism of action of different hormonal therapies in AR signaling pathway. * First generation nonsteroidal AR antagonist.

The AR is a nuclear receptor and a ligand-dependent transcription factor that regulates the expression of certain specific genes, including PSA. Its most potent native ligand is 5α-dihydrotestosterone (DHT) that is generated by intracellular metabolism of testosterone, an endogenous androgen synthesized primarily in the testes. The DHT-AR binding drives the AR translocation from cytoplasm to cell nucleus where the AR forms dimer and binds to the androgen response elements in DNA. Co-activators (coregulatory proteins) are then recruited to boost transcription, leading to prostate cancer cell proliferation and survival [2,3]. As shown in Figure 5, castration therapies (both surgical or medical) and abiraterone acetate block the androgen production, while AR antagonists restrain the AR function through competitively binding to the androgen binding site of AR in cell cytoplasm. Intriguingly, enzalutamide, apalutamide, and darolutamide mechanistically distinguish themselves from the first generation nonsteroidal AR antagonists by interfering several stages in the AR signaling pathway [14,23,24]. In addition to competitively suppressing androgen-AR binding, these second-generation nonsteroidal AR antagonists also inhibit the AR translocation from cytoplasm to cell nucleus, the coactivator recruitment, and the AR-DNA binding.

## 6. Strategies to Overcome the Resistance to the Second-Generation AR Antagonists

In spite of the above-mentioned therapeutic benefits of three second-generation AR antagonists in patients with mCRPC, nmCRPC, or mCSPC, a considerable portion of patients are primarily resistant to the treatment. As summarized in Table 10, 63% of the patients with mCRPC after treating with enzalutamide are still at risk of death; 28%–41% of the patients with nmCRPC under the treatment of enzalutamide, apalutamide, or darolutamide, in combination with ADT, are on the line of progression to metastasis or death; 31%–48% of patients of mCSPC under the treatment of enzalutamide or apalutamide are exposed to radiographic progression or death. Additionally, acquired resistance emerges with the time of treatment.

### 6.1. Mechanisms of the Resistance to the Second-Generation AR Antagonists

**Figure 6:**
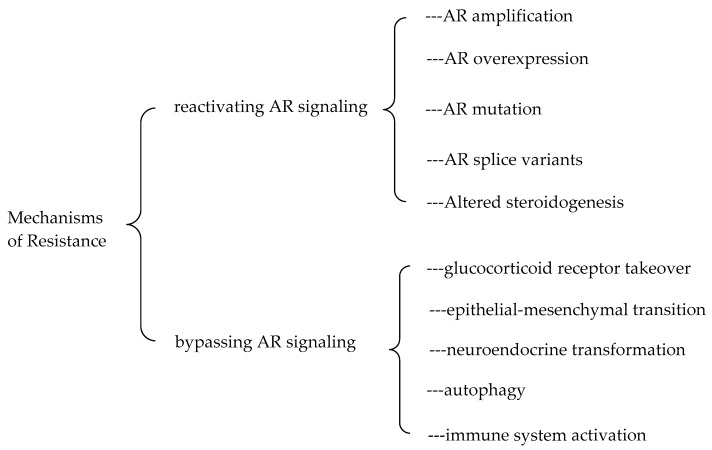
Mechanisms of resistance to the second-generation AR antagonists.

The accurate mechanisms of the resistance to the second-generation AR antagonists are still not very clear. Several proposed mechanisms underlying the resistance to the second-generation AR antagonists are summarized in Figure 6. These mechanisms can be classified into two categories: reactivating androgen receptor signaling and bypassing androgen receptor signaling [67]. The reactivation of AR signaling can be achieved by AR amplification and AR overexpression, AR gain-of-function mutations, spliced AR variants (e.g., AR-V7), and intramolecular generation of androgens. The resistance can also be gained through bypassing AR signaling pathway including glucocorticoid receptor takeover, epithelial-mesenchymal transition, neuroendocrine transformation, autophagy, and immune system activation. More details about these proposed mechanisms have been comprehensively reviewed in the literature [67,68,69,70].

### 6.2. Strategies to Overcome the Resistance to the Second-Generation AR Antagonists

#### 6.2.1. Combination Therapy

Combination therapy through targeting multiple complementary mechanisms of action has been recognized in recent years to be a promising strategy to overcome drug resistance [71]. Taking advantage of currently available therapies for CRPC, development of their optimal combinations as multifaceted therapies emerges as one of research hotspots in the field. Additionally, numerous of enzalutamide-based combinations are currently under clinical investigation at different phases, most of which have been tabulated by Tucci et al. in their review article [67]. With the goal to sensitize enzalutamide, these combinations were designed based on the current proposed mechanisms of resistance to the second-generation AR antagonists, as listed in Figure 6. For example, the CORT125281 (glucocorticoid receptor antagonist, NCT03437941), metformin (induces epithelial-mesenchymal transition via suppression of transforming growth factor beta 1/signal transducer and activator of transcription 3 (TGF-β1/STAT3) pathway, NCT02339168 and NCT02640534), galunisertib (TGF-*β* inhibitor, NCT02452008), AZD5363 (protein kinase B (AKT) inhibitor, NCT0331054), pembrolizumab (an anti-PD-1 checkpoint, NCT02861573 & NCT02787005), and AZD5069 (chemokine receptor antagonist, NCT03177187) are currently in clinical studies in combination with enzalutamide.

#### 6.2.2. Target AR with Other Strategies

AR is a nuclear steroid receptor that comprises three functional domains including the ligand-binding domain (LBD, *C*-terminal end), the DNA-binding domain (DBD, central portion), and the transactivation domain (NTD or TAD, *N*-terminal end) [3]. Enzalutamide, apalutamide, and darolutamide competitively bind to the ligand-binding pocket of the LBD and inhibit the agonistic action of intrinsic ligands. The compounds that still target AR but can overcome the resistance of the second-generation AR antagonists include new LBD-targeted AR antagonists with novel chemical scaffolds, TAD (or NTD)-targeted AR antagonists, DBD-targeted AR antagonists, and AR degraders.

New AR antagonists that still bind to the LBD but with distinct chemical structures can overcome the resistance due to the point mutations, which has been exemplified by the successful story of darolutamide [72]. As abovementioned, darolutamide was developed targeting enzalutamide-resistant prostate cancer. It was revealed to suppress the transcriptional activity of some AR mutants including T878G that was responsible of converting enzalutamide into a partial AR agonist. Recently, halogen-substituted anthranilic acid derivatives have been established as a new chemical scaffold that inhibits the transactivation of both wild-type AR and AR mutants that render treatment resistance to the first-generation and second-generation nonsteroidal AR antagonists [73].

Several compounds that target the TAD or DBD of the AR have been demonstrated to possess potential in treating CRPC, which have been comprehensively summarized in an excellent review article [3]. The EPI compounds that were first isolated from marine sponges and derived from bisphenol A represent the most well-established inhibitors of AR-TAD. This group of compounds down-regulates the expression of full length AR and truncated AR variants (e.g., AR-V7) through suppressing tau-1 (transcriptional activation unit 1) and tau-5 of the TAD [74]. They inhibited AR-positive prostate cancer cell proliferation in both in vitro and in vivo experiments and suppressed the growth of AR-positive prostate cancer cell-derived tumors. The most developed EPI compound, EPI-506, has advanced to a Phase I/II clinical trial (NCT02606123) in patients with mCRPC after enzalutamide and/or abiraterone treatment.

Hairpin polyamide was developed as an AR antagonist that directly inhibits AR binding to DNA and blocks the transcription processes mediated by AR [75]. Hairpin polyamide compounds may be a good strategy to overcome the resistant to the second-generation AR antagonists because they target the transcription driven by both AR and glucocorticoid receptor. Moreover, overexpression of glucocorticoid receptor has been proposed to be one of the critical pathways leading to the resistance of the second-generation AR antagonists.

A new strategy to combat the resistance of the second-generation AR antagonists is to target AR protein for degradation, which is a completely different mechanism compared with those for AR antagonists. Additionally, AR degradation has been reported to be a likely prerequisite for prostate cancer tumor shrinkage based on an in vivo experiment [76]. AR degradation via proteolysis-targeting chimeras (PROTACs) is currently the most intriguing development in this field because this technology replaces the occupancy-driven mechanism for AR antagonist with an event-driven mechanism [77,78]. AR-PROTACs are bifunctional chimeras that can bring AR protein and the E3 ubiquitin ligase in close proximity, resulting in AR ubiquitination and subsequent degradation. AR-PROTACs have been verified in vitro and in vivo to be a better therapeutic strategy than AR antagonists for targeting AR signaling [79]. ARV-110 is the first AR-PROTAC to enter a phase I clinical trial (NCT03888612) in 2019. This clinical study aims to evaluate the pharmacokinetics, safety, and tolerability of ARV-110 in patients with mCRPC who have received more than two systemic therapies.

Additionally, the association between metformin (an antidiabetic drug) and reduced prostate cancer risk in patients with type 2 diabetics prompted a plethora of investigations on the therapeutic effects of metformin. Metformin is undergoing several clinical trials for prostate cancer, which has been summarized in the literature [80]. The mechanism of action underlying its anti-prostate cancer activity has been extensively explored [80], with the crosstalk between adenosine monophosphate-activated protein kinase (AMPK) activation and AR degradation as the most attractive one [81]. It may therefore be a new strategy, especially for prostate cancer patients with diabetics, to use AMPK activators to overcome the resistance to second-generation nonsteroidal AR antagonists. In addition, cyclin-dependent protein kinase 9 (CDK9) is a druggable target for prostate cancer because CDK9 can not only phosphorylate AR and activate AR transcriptional activity but also target anti-apoptotic proteins [82]. Therefore, CDK9 inhibitors may serve as a better therapeutic strategy over the second-generation nonsteroidal AR antagonists for the patients with CRPC.

## 7. Conclusions

In conclusion, an interdisciplinary collaboration led the discovery of enzalutamide as the first second-generation nonsteroidal androgen receptor (AR) antagonist with a strong binding affinity to AR. Enzalutamide can significantly prolong not only overall survival time and metastatic free survival time for patients with lethal CRPC, but also castration resistant free survival time for patients with CSPC. Enzalutamide has thus been approved by the US Food and Drug Administration (FDA) for the treatment of both metastatic (in 2012) and non-metastatic (in 2018) CRPC, as well as CSPC (2019) on the basis of the therapeutic benefits observed from AFFIRM, PREVAIL, PROSPER, and ARCHES Phase III clinical trials. Encouraged by the positive clinical results of enzalutamide, two other second-generation AR antagonists, apalutamide, and darolutamide have recently been approved by the FDA for the treatment of prostate cancer. These three second-generation AR antagonists not only offer patients with different stages of prostate cancer with alternative therapeutics, but also verified that AR signaling pathway plays a pivotal role in the progression of both CSPC and CRPC. Several approaches have been developed for the syntheses of these three second-generation AR antagonists, with three main strategies for the syntheses of the core structure of enzalutamide and apalutamide. The drawback of these AR antagonists as therapeutics for prostate cancer is the drug resistance, which can be developed by reactivating or bypassing androgen receptor signaling pathway. Combination therapies taking advantage of multiple complementary mechanisms of action and targeting AR with other mechanisms may serve as good strategies to overcome the resistance.

## Figures and Tables

**Table 1:** Current Treatments with Survival Benefit for Patients with Prostate Cancer.

Brand Name	Generic Name	Approval Date	Treatments	Category
Taxotere	Docetaxel in combination with prednisone	19 May 2004	mCRPC	Chemotherapy
Jevtana	Cabazitaxel in combination with prednisone	17 June 2010	mCRPC after docetaxel	Chemotherapy
Xofigo	radium-223	15 May 2013	mCRPC	Radiotherapy
Provenge	Sipuleucel-T	29 April 2010	Asymptomatic or minimally symptomatic mCRPC	Immunotherapy
Zytiga	Abiraterone acetate in combination with prednisone	28 April 2011	mCRPC after docetaxel	Hormonal therapy
Zytiga	Abiraterone acetate in combination with prednisone	10 December 2012	mCRPC before chemotherapy	Hormonal therapy
Zytiga	Abiraterone acetate in combination with prednisone	7 February 2018	mCSPC	Hormonal therapy
Erleada	Apalutamide	14 February 2018	nmCRPC	Hormonal therapy
Erleada	Apalutamide	17 September 2019	mCSPC	Hormonal therapy
XTANDI	Enzalutamide	31 August 2012	mCRPC after docetaxel	Hormonal therapy
XTANDI	Enzalutamide	13 July 2018	nmCRPC	Hormonal therapy
NUBEQA	Darolutamide	30 July 2019	nmCRPC	Hormonal therapy
XTANDI	Enzalutamide	16 December 2019	mCSPC	Hormonal therapy

nmCRPC: non-metastatic castration-resistant prostate cancer. mCRPC: metastatic castration-resistant prostate cancer mCSPC: metastatic castration-sensitive prostate cancer.

**Table 2:** FDA-approved second-generation AR antagonists for prostate cancer.

Brand Name	Generic Name	mCRPC	nmCRPC	mCSPC
XTANDI	enzalutamide	yes	yes	yes
Erleada	apalutamide	no	yes	yes
NUBEQA	darolutamide	no	yes	no

nmCRPC: non-metastatic castration-resistant prostate cancer. mCRPC: metastatic castration-resistant prostate cancer. mCSPC: metastatic castration-sensitive prostate cancer.

**Table 3:** Major therapeutic benefits brought by enzalutamide based on AFFIRM.

Enzalutamide AFFIRM Phase III Trial (NCT00974311)			
End Points	Enzalutamide (n = 800)	Placebo (n = 399)	Hazard Ratio (95% CI)
**Primary End Points**			
Median OS (mo)	18.4	13.6	0.63
**Secondary End Points**			
Median time to rPFS (mo)	8.3	2.9	0.4
Median time to first SRE (mo)	16.7	13.3	0.69
Median time to PSA progression (mo)	8.3	3	0.25
* PSA response (%) of no.			
decline ≥90% from baseline	25 (731)	1 (3300	
decline ≥50% from baseline	54 (731)	1 (330)	
Serious AEs (%)	39.9	38.8	

OS: Overall Survival; rPFS: Radiographic Progression-free Survival; SRE: Skeletal-related Event; PSA: Prostate-specific Antigen; AEs: Adverse Events. * Does not include patients who discontinued or died during the trial.

**Table 4:** Major therapeutic benefits brought by enzalutamide based on PREVAIL.

Enzalutamide PREVAIL Phase III Trial (NCT01212991)			
End Points	Enzalutamide (n = 872)	Placebo (n = 845)	Hazard Ratio (95% CI)
**Primary end point**			
Median OS (mo)	18.4	13.6	0.63
Median time to rPFS (mo)		3.9	0.19
**Secondary end points**			
Median time to first SRE (mo)	16.7	13.3	0.69
Median CC initiation time (mo)	28	10.8	0.35
Median time to PSA progression (mo)	11.2	2.8	0.17
* PSA response (%) of no.			
decline ≥ 90% from baseline	47 (854)	1 (777)	
decline ≥ 50% from baseline	78 (854)	3 (777)	
* Serious AEs (%) of no.	44.1 (871)	3.5 (844)	

OS: Overall Survival; rPFS: Radiographic Progression-free Survival; SRE: Skeletal-related Event; CC: Cytotoxic Chemotherapy; PSA: Prostate-specific Antigen; AEs: Adverse Events. * Does not include patients who discontinued or died during the trial.

**Table 5:** Major therapeutic benefits brought by enzalutamide based on PROSPER.

Enzalutamide PROSPER Phase III Trial (NCT02003924)			
End Points	Enzalutamide (n = 933)	Placebo (n = 468)	Hazard Ratio (95% CI)
**Primary end point**			
Median MFS (mo)	36.6	14.7	0.29
Median time to rPFS (mo)		3.9	0.19
**Secondary end points**			
Median OS (mo)			
Median time to C-FS (mo)	38.1	34	
Median first time use of CC (mo)		39.7	0.38
Median time to PP (mo)	18.5	18.4	0.96
Median time to PSA progression (mo)	37.2	3.9	0.07
* PSA response (%)			
decline ≥ 90% from baseline	55.9	0.4	
decline ≥ 50% from baseline	76.3	2.4	
decline to undetectable level	9.6	0	
* Serious AEs (%) of no.	24.3 (930)	18.9 (465)	

MFS: Metastasis Free Survival; rPFS: Radiographic Progression-free Survival; OS: Overall Survival; C-FS: Chemotherapy-Free Survival; PP: Pain Progression; CC: Cytotoxic Chemotherapy; PSA: Prostate-specific Antigen; AEs: Adverse Events; * Does not include patients who discontinued or died during the trial.

**Table 6:** Major therapeutic benefits brought by enzalutamide based on ARCHES.

Enzalutamide ARCHES Phase III Trial (NCT02677896)			
End Points	Enzalutamide + ADT (n = 574)	Placebo + ADT (n = 576)	Hazard Ratio (95% CI)
**Primary End Point**			
^‡^ Median time to rPFS based on ICR via PCWG2 (mo)		19.4	0.39
^†^ Median time to rPFS based on ICR via PAC (mo)		19.0	0.39
**Secondary End Points**			
Median OS (mo)			0.19
Time to NAT	30.2		0.28
Median time to CR (mo)		13.9	0.28
PSA undetctable rate (%) of no.	68.1 (511)	17.6 (506)	
* Serious AEs (%) of no.	18.2 (572)	19.5 (574)	

rPFS: Radiographic Progression-free Survival; OS: Overall Survival; NAT: New Antineoplastic Therapy; CR: Castration resistance; PSA: Prostate-specific Antigen; AEs: Adverse Events; ^‡^ Radiographic Progression-Free Survival (rPFS) Based on Independent Central Review (ICR) of Bone Scan According to Prostate Cancer Clinical Trials Working Group 2 (PCWG2) Criteria; ^†^ rPFS Based on ICR of Bone Scan According to Protocol Assessment Criteria; * Does not include patients who discontinued or died during the trial.

**Table 7:** Major therapeutic benefits brought by apalutamide based on SPARTAN.

Apalutamide SPARTAN Phase III Trial (NCT01946204)			
End Points	Apalutamide (n = 806)	Placebo (n = 401)	Hazard Ratio (95% CI)
**Primary End Points**			
Median MFS (mo)	40.5	16.2	0.28
**Secondary End Points**			
Median time to metastasis (mo)	40.5	16.6	0.28
Median time to PFS (mo)	40.5	14.7	0.29
Median OS (mo)		39.0	
Median time to CC (mo)			
* Serious AEs (%) of no.	24.8 (803)	23.1 (398)	

MFS: Metastasis Free Survival; PFS: Progression Free Survival; OS: Overall Survival; CC: Cytotoxic Chemotherapy; AEs: Adverse Events; * Does not include patients who discontinued or died during the trial.

**Table 8:** Major therapeutic benefits brought by apalutamide based on TITAN.

Apalutamide TITAN Phase III Trial (NCT02489318)			
End Points	Apalutamide + ADT (n = 525)	Placebo + ADT (n = 527)	Hazard Ratio (95% CI)
**Primary End Points**			
OS (% alive)	82..4	73.5	0.67
rPFS (%)	68.2	47.5	0.48
**Secondary End Points**			
Median time to PSA progression (mo)		12.9	0.26
* Serious AEs (%) of no.	19.2 (574)	20.3 (527)	

OS: Overall Survival; rPFS: Radiographic Progression-free Survival; PSA: Prostate-specific Antigen; AEs: Adverse Events; * Does not include patients who discontinued or died during the trial.

**Table 9:** Major therapeutic benefits brought by darolutamide based on ARAMIS.

Darolutamide ARMIS Phase III Trial (NCT02200614)			
End Points	Darolutamide (n = 955)	Placebo (n = 554)	Hazard Ratio (95% CI)
**Primary End Point**			
Median MFS (mo)	40.4	18.4	0.41
**Secondary End Points**			
Median OS (mo)			0.71
Median first time use of CC (mo)		38.2	0.43
Median time to PP (mo)	40.3	25.36	0.65
Median time to SSE (mo)			0.43
* Serious AEs (%) of no.	24.8 (954)	10.5 (554)	

MFS: Metastasis Free Survival; OS: Overall Survival; PP: Pain Progression; SSE: Symptomatic Skeletal Event; AEs: Adverse Events; * Does not include patients who discontinued or died during the trial.

**Table 10:** Hazard Ratios for the primary end points in the phase III trials of three AR antagonists.

Trial Name	AR Antagonist	Primary End Point	Hazard Ratio (95% CI)	Patients
AFFIRM	enzalutamide	Median overall survival	0.63	mCRPC
PROPSER	enzalutamide	Median metastasis-free survival	0.29	nmCRPC
SPARTAN	apalutamide	Median metastasis-free survival	0.28	nmCRPC
ARAMIS	darolutamide	Median metastasis-free survival	0.41	nmCRPC
ARCHES	enzalutamide	Median rPFS	0.31	mCSPC
TITAN	apalutamide	Median rPFS	0.48	mCSPC
